# Slider versus Likert scales: Psychometric properties in ambulatory assessment

**DOI:** 10.3758/s13428-026-02992-4

**Published:** 2026-03-30

**Authors:** Dominik Vollbracht, Charlotte Ottenstein, Sabrina Ecker, Tanja Lischetzke

**Affiliations:** RPTU University Kaiserslautern-Landau, Fortstraße 7, 76829 Landau, Germany

**Keywords:** Slider scales, Visual analogue scales, Likert scales, Ambulatory assessment, Experience sampling, Intensive longitudinal data

## Abstract

Slider scales, a type of visual analogue scale, are commonly used as a response format in smartphone-based ambulatory assessment studies. This may be due to several advantages of slider scales, including the use of a metric scale compared to an ordinal Likert scale and the intuitive use of slider scales on touchscreens. However, research on the comparability of Likert and slider scales remains scarce, especially in the context of ambulatory assessment (i.e., intensive longitudinal data). To address this gap, we conducted a 3-week ambulatory assessment study with four measurement occasions per day, in which we experimentally manipulated the response format (Likert vs. slider scales) between groups. Our final sample consisted of 21,730 measurement occasions nested in 406 participants. We tested for measurement invariance across response-format groups and compared their psychometric properties, including reliability, within-person variability, and validity. Results indicated measurement invariance across groups, with equal factor loadings at the within-person level and equal factor loadings, intercepts, and indicator-specific residual variances at the between-person level. In addition, we found no significant differences in reliability or validity and only minor differences in within-person variability. We discuss the implications of these findings for the design of ambulatory assessment studies and offer recommendations for future research.

## Introduction

The choice of response format is a critical decision when designing a study or constructing a test in the behavioral and social sciences. One of the most commonly used response formats is the Likert scale, which consists of a discrete ordinal rating scale (Joshi et al., 2015). Another popular response format is the slider scale (or visual analogue scale). Unlike Likert scales, slider scales provide a continuous response format, theoretically allowing for a more fine-grained measurement of the construct of interest. Slider scales are now widely used in various types of assessment, particularly in ambulatory assessment studies (e.g., Cloos et al., 2023; He et al., 2022; Hoemann et al., 2021; Le et al., 2021).

Ambulatory assessment is an umbrella term that encompasses various methods, including experience sampling, ecological momentary assessment, and daily diary studies (e.g., Trull & Ebner-Priemer, 2014). By collecting data repeatedly in participants’ natural environments, ambulatory assessment studies capture time-varying psychological constructs such as behavior or emotions. Because of this real-time assessment, measures are less susceptible to retrospective bias (Bolger & Laurenceau, 2013; Hamaker & Wichers, 2017; Trull & Ebner-Priemer, 2013). These advantages make ambulatory assessments a valuable tool for capturing within-person dynamics and interindividual differences in these dynamics.

When designing an ambulatory assessment study, researchers must make several methodological decisions. While the effects of some design choices, such as sampling intensity, on the psychometric properties of measurement instruments have been studied (e.g., Hasselhorn et al., 2022), the effects of others remain underexplored. This includes the choice of response format. Slider scales are implemented in many popular ambulatory assessment software platforms, including movisens (movisens GmbH, 2024), SEMA3 (O’Brien et al., 2024), LifeData (LifeData, 2024), or m-path (Mestdagh et al., 2023). They have been used in numerous ambulatory assessment studies across diverse research areas, including affect and emotion research (de Leersnyder et al., 2018; e.g., Dejonckheere et al., 2018; Grommisch et al., 2020), health psychology (e.g., Kamarck et al., 2012; Kraiss et al., 2022; Nap-van der Vlist et al., 2021), work psychology (e.g., Hernandez et al., 2023; Suwa et al., 2024; Tump et al., 2022), and personality psychology (e.g., Edmondson et al., 2013; van Halem et al., 2024). Their widespread use is likely attributable to several perceived advantages in ambulatory assessment.

### Characteristics of slider scales

A slider scale is a rating scale in which participants set and adjust a marker along a horizontal or vertical continuum to indicate their response (Reips & Funke, 2008). Many slider scales have 100 or more steps, which theoretically allows for higher measurement resolution than Likert scales. The endpoints of the scale are labeled with anchors that define the extremes of the measured variable and provide clear reference points for respondents (e.g., *fully disagree* to *fully agree*). Slider scales originate from visual analogue scales (the terms are often used interchangeably), which have been used in pen-and-paper surveys for several decades, particularly in medical contexts such as pain assessment (Langley & Sheppeard, 1985). In some applications, slider scales contain ticks along the continuum, which can influence response distributions by acting as additional anchors (Matejka et al., 2016). With the increasing use of smartphones in ambulatory assessment studies (Trull & Ebner-Priemer, 2014), slider scales have become a viable response format, as participants can intuitively adjust them using the device’s touchscreen. To evaluate their psychometric properties, slider scales have been compared with Likert scales in various studies. Recent evidence also shows that digital slider scales yield highly precise and consistent responses even when operated on touchscreens, alleviating concerns about finger–screen coordination errors in ambulatory assessment settings. In a large-scale study (Cloos et al., 2025), it was found that participants could reproduce specific target values with high accuracy and demonstrated stable interpretations of midpoint positions across measurement occasions.

### Previous research on the properties of slider versus Likert scales

Several studies have compared the descriptive statistics of slider and Likert scales in cross-sectional surveys using randomized experimental designs. In web-based settings, slider scales demonstrated longer response times and lower item-level compliance than Likert scales (Cook et al., 2001; Couper et al., 2006; Funke, 2016; Sikkel et al., 2014). However, findings on item response distributions are mixed: Some studies found no significant differences (Couper et al., 2006; Kuhlmann et al., 2017), whereas Funke (2016) reported significant differences in the response distributions between the two response formats, with fewer responses at the midpoint of the slider scale. The author attributed these differences to the use of an initial midpoint position for the slider, which was not implemented in the other studies.

Only a few studies have compared the psychometric properties of measurement instruments using the two response formats. García-Pérez (2023) found that the intervals between the response categories of a Likert scale were not directly comparable to the corresponding intervals on a slider scale. For example, the distance between 1 and 2 on a five-point Likert scale was not equivalent to the distance between 0 and 20 on a 0–100 slider scale. However, this discrepancy did not result in differences in the psychometric properties of the two response formats (García-Pérez, 2023; Kuhlmann et al., 2017). Similarly, Simms et al. (2019) found no significant differences in reliability (internal consistency and test–retest comparisons), criterion validity, or descriptive statistics between Likert and slider scales. Thus, when examining psychometric properties in cross-sectional surveys, neither response format appears to offer a clear advantage over the other.

Only recently have studies begun to compare slider and Likert scales in ambulatory assessment designs. Overall, these investigations indicate that the two response formats perform similarly in terms of reliability, compliance, and descriptive statistics (e.g., Businelle et al., 2024). In a direct experimental comparison, Haslbeck et al. (2025) found largely equivalent results across response formats in a 14-day ambulatory assessment study, with slider scales yielding slightly higher within-person correlations and somewhat stronger associations with measures of psychopathology. Taken together, these findings suggest that both response formats are generally suitable for intensive longitudinal research, with only minor practical or psychometric differences between them.

### The present research

Although previous research has provided valuable insights into the characteristics of slider versus Likert scales, several limitations remain. First, only a small number of recent studies have examined these response formats in the context of intensive longitudinal and ambulatory assessment designs. Second, most existing investigations relied on web-based data collected via mouse-and-keyboard interfaces, whereas the psychometric properties of smartphone-based slider scales (i.e., scales implemented using a touchscreen) remain less well understood. Third, most previous studies assessed psychometric properties using manifest variable models that do not account for measurement error. Consequently, little is known about the psychometric properties (reliability, validity) of slider versus Likert scales within a latent variable framework.

The present research aimed to address these gaps by comparing slider and Likert scales in terms of measurement invariance, within-person and between-person reliability, within-person variability, and within-person relationships with other constructs (i.e., validity estimates). As time-varying (latent) constructs, we focused on momentary pleasant–unpleasant mood and two meta-mood constructs (momentary attention to and clarity of negative emotions), which were measured with multiple items each. Additionally, we measured momentary stress as a manifest covariate. In the following sections, we describe the psychometric properties of interest and outline our research questions (RQ).

At the time of preregistration, no ambulatory assessment studies directly comparing Likert and slider formats were available. Therefore, we preregistered the study without directional hypotheses regarding the psychometric properties of the two response formats.

#### Measurement invariance

Measurement invariance is essential for comparing self-reported measures across groups, time points, and experimental conditions (Maassen et al., 2023). It reflects the extent to which a measurement model remains consistent and a latent variable is measured equivalently across conditions (e.g., different response formats). For example, to compare latent means and covariances between Likert and slider scales, at least strong measurement invariance must be established. This level of invariance requires that factor loadings and intercepts are equivalent across response formats (Meredith, 1993). Therefore, we aimed to assess the degree of measurement invariance between the two response formats. As intensive longitudinal data represent a form of multilevel data, with measurement occasions nested within participants, we focused on both within-person and between-person measurement invariance using a two-level confirmatory factor analysis approach.

- *RQ 1*: When used to assess time-varying constructs, do the two response formats (Likert vs. slider scales) show measurement invariance at the within- and between-person level (i.e., equality of measurement model parameters across formats)?

#### Reliability

Castro-Alvarez et al. (2025) reviewed methodological approaches for estimating reliability in intensive longitudinal data and categorized them into different methodological frameworks. Based on their recommendation that researchers apply multiple frameworks and compare the resulting estimates, we implemented two complementary approaches to examine potential differences in reliability across response formats.

Within the multilevel confirmatory factor analysis (CFA) framework, reliability is partitioned into within-person and between-person components (Geldhof et al., 2014). Within-person reliability reflects the proportion of within-person variance in item responses attributable to the within-person latent factor, whereas between-person reliability captures the proportion of expected person-level variance attributable to the between-person latent factor. To address potential overestimation due to unaccounted measurement error, we employed the updated approach proposed by Lai (2021), which extends the original formulation by Geldhof et al. (2014).^1^

To further follow the recommendation by Castro-Alvarez et al. (2025), we additionally applied the two-level random dynamic model (2RDM) proposed by Xiao et al. (2023). This approach is based on the dynamic structural equation modeling (DSEM) framework, which allows for variation in loadings, residual variances, and dynamic effects across individuals (Asparouhov et al., 2018). We used the 2RDM to estimate within-person reliability separately for each individual.

- *RQ 2*: Do the two response formats differ in within-person and between-person reliability for the time-varying constructs being assessed?

#### Within-person variability

Within-person variability describes the extent to which a time-varying construct fluctuates around an individual’s mean over time. It is essential for the analysis of within-person dynamics (Heck & Thomas, 2015; Hox, 2002). Moreover, if within-person variability is too low, relationships between time-varying variables should not be analyzed (Podsakoff et al., 2019). Therefore, it is important to examine whether the choice of response format (slider or Likert scale) affects within-person variability. If reliability is roughly equivalent across response formats, and within-person item variances are comparable, as observed by Haslbeck et al. (2025), then no meaningful differences in within-person variability would be expected between slider and Likert scales.

- *RQ 3*: Do slider and Likert scales differ in within-person variability?

#### Relationships with other time-varying variables (validity estimates)

As an indicator of criterion validity, we aimed to test whether the within-person relationships among time-varying constructs are the same across response formats. Specifically, we compared the covariances of the latent variables of interest (momentary pleasant–unpleasant mood, momentary attention to and clarity of negative emotions) with a single-item measure of momentary stress. Additionally, we compared the covariances among the latent constructs themselves. As Haslbeck et al. (2025) found higher absolute correlations between time-varying variables for slider scales, we would expect the same pattern here.

- RQ 4: Do the two response formats differ in the relationships between the time-varying constructs of interest and other time-varying constructs (i.e., validity estimates)?

#### Other outcomes

In addition to the preregistered research questions, we explored potential differences between response formats in several additional outcomes examined by Haslbeck et al. (2025). Specifically, we compared bivariate correlations among constructs, means, and within-person standard deviations across the two response formats.

## Method

### Study design

The study consisted of an initial online survey (assessing demographic variables and trait measures) and an ambulatory assessment phase (measuring time-varying variables), which consisted of four measurement occasions per day for 21 days. For the ambulatory assessment phase, participants chose one of two schedules (09:00–21:00 or 10:30–22:30) that best fit their waking hours. Participants were randomly assigned to one of two experimental conditions. For a subset of items (specifically those assessing momentary mood, attention to negative emotions, and clarity of negative emotions), the response format was experimentally manipulated. In the Likert scale group, these items were presented in a Likert scale format, whereas in the slider scale group, the same items were presented in a slider scale format. Momentary stress as a covariate was assessed with a Likert scale in both groups.^2^ All other constructs (e.g., emotions, emotion regulation strategies, and physical activity) were assessed with a Likert scale in both groups. The study concluded with a final online survey (measuring additional trait measures).

### Participants

To take part in the study, participants had to be at least 18 years old and have a smartphone (iOS or Android) with internet access. They were recruited through social media, flyers, posters, and emails. In total, 421 participants started the ambulatory assessment phase after being randomly assigned to one of the two experimental groups, resulting in 22,645 measurement occasions.^3^ After data cleaning (see Data cleaning section), the final sample consisted of 21,730 measurement occasions nested in 406 participants (Likert scale group = 10,640 measurement occasions nested in 200 participants; slider scale group = 11,090 measurement occasions nested in 206 participants). On average, participants completed 53.52 measurement occasions (64%) out of 84 possible (*SD* = 25.46; range, 1–84). Regarding demographics, 78% of participants identified as female, 21% as male, and 1% as diverse or inter. Age ranged from 18 to 68 years (*M* = 27.40; *SD* = 9.19).

### Procedure

After registration, participants were randomly assigned to one of the two experimental conditions (Likert scales group or slider scales group). Participants were then emailed a URL for the initial online survey. After completing the survey, participants received instructions to download and install the smartphone application SEMA3 (O’Brien et al., 2024). Through the app, participants received four notifications per day according to their chosen schedule. After each notification, participants had 65 min to complete the corresponding questionnaire via the app. After the 3-week ambulatory assessment phase, participants were emailed the URL for the final online survey.

Participants could receive personalized feedback on their responses. In addition, they were financially compensated for their participation based on their compliance during the ambulatory assessment phase. Undergraduate psychology students were able to substitute financial compensation for course credit. Minimum compliance of 50% was required to receive €15, while a minimum of 80% was required to receive €40. The study was approved by the Psychological Ethics Committee of the RPTU University Kaiserslautern-Landau (formerly University of Koblenz-Landau), Germany. The procedures used in this study adhered to the tenets of the Declaration of Helsinki.

### Data cleaning

Two data cleaning steps were performed. First, measurement occasions with missing responses on any items of interest (i.e., those aborted before all relevant items were answered) were excluded. This resulted in a sample of 22,093 measurement occasions nested within 406 participants. Possible reasons for nonresponse included participants forgetting to respond to prompts, being interrupted during the assessment, or experiencing technical issues with the app. These occasions were excluded to ensure that analyses were based on complete data for all items of interest.

Second, to identify careless responding, we examined inconsistency across reverse-scored momentary mood items following the approach developed by Hasselhorn et al. (2025).^4^ A total of 363 measurement occasions were excluded due to inconsistent responding, resulting in a final sample of 21,730 measurement occasions nested within individuals.

### Measures

During the ambulatory assessment phase, we measured our constructs of interest (momentary pleasant–unpleasant mood, attention to and clarity of negative emotions), a manifest covariate (momentary stress), and additional constructs not relevant to the present research. The full list of items can be found in the codebook in the OSF repository (see Open Practices Statement section).

#### Operationalization of response formats

**Fig. 1:**
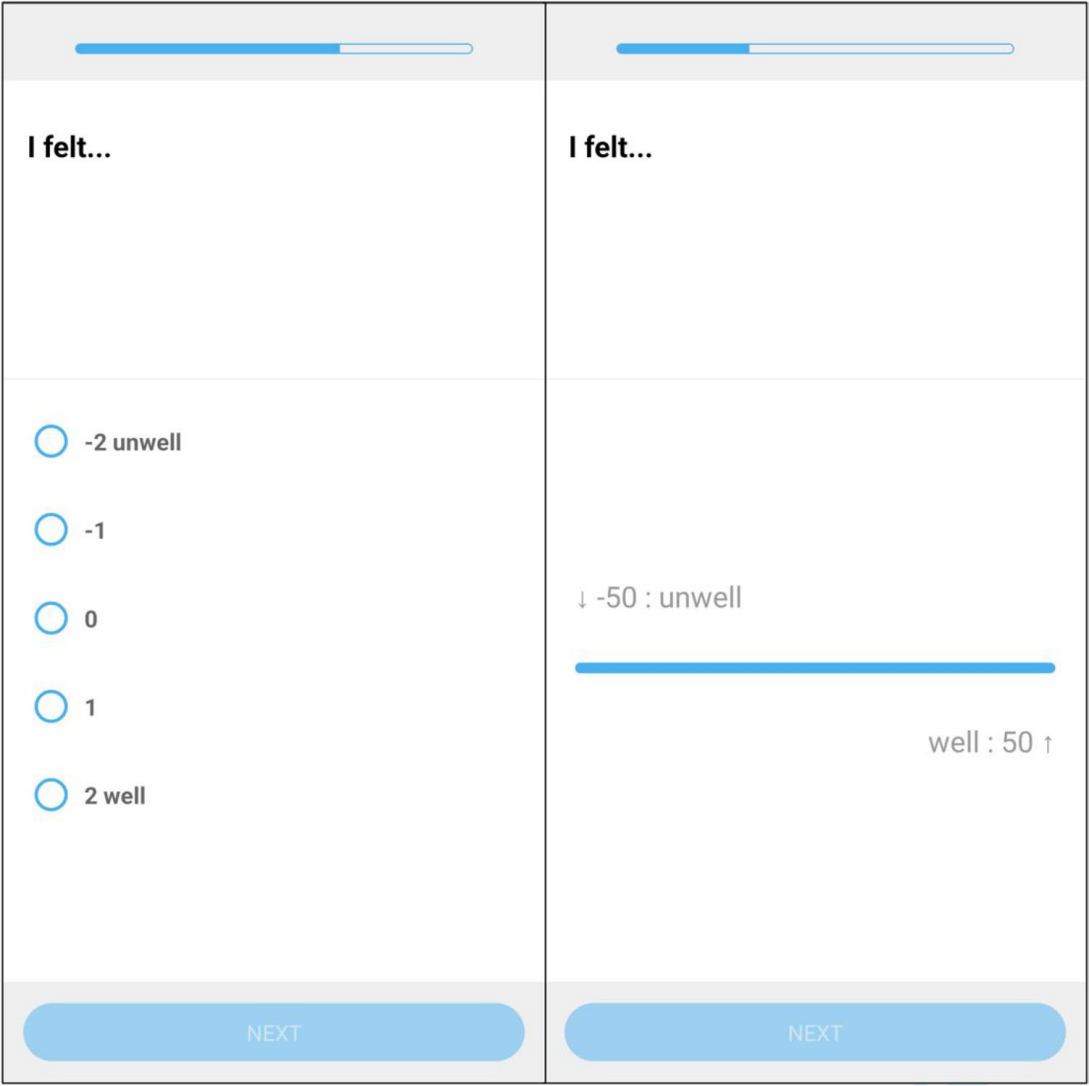
Visualization of both response formats in the SEMA3 app. *Note*. Screenshots of a translated item assessing momentary mood using a Likert scale (left) and a slider scale (right) in the SEMA3 app

We used a 101-point slider scale, a commonly applied range in research, with values from − 50 to 50 for bipolar momentary pleasant–unpleasant mood items (e.g., − 50/*unwell*, 50/*well*) and from 0 to 100 for unipolar attention to and clarity of negative emotions items (0/*does not apply at all*, 100/*fully applies*). We did not use a starting position or ticks, as previous research has shown that this can affect response distributions (Funke, 2016; Matejka et al., 2016). Both extremes were labeled with the respective word/statement pairs, and participants saw only the numbers at the extremes (e.g., 0 and 100), but not the number they chose. For the Likert scales, we used a five-point scale (− 2 to 2 for the bipolar mood items and 1 to 5 for the attention to and clarity of negative emotions items), for three reasons. First, we wanted to have a midpoint, as slider scales provide a relative midpoint. Second, five response options fit on most mobile phone screens without scrolling. Finally, a five-point scale is one of the most commonly used Likert scale formats (Kusmaryono et al., 2022). Only the extremes were labeled with word/statement pairs (e.g., 1/*does not apply at all*, 5/*fully applies*), while the intermediate response options were labeled with numbers only. A visual representation of both response formats is provided in Fig. 1.

#### Momentary pleasant–unpleasant mood

Momentary mood was assessed with an adapted short version of the Multidimensional Mood Questionnaire (Steyer et al., 1997), which has been used in previous ambulatory assessment studies (e.g., Lischetzke et al., 2012, 2022). The instruction read, “How did you feel just now (right before this prompt)? I felt…” Pleasant–unpleasant mood was measured by four bipolar items: *unwell–well* (pum1), *good–bad* (pum2) [reverse-scored], *dissatisfied–satisfied* (pum3), and *happy–unhappy* (pum4) [reverse-scored]. The poles of the response format were labeled with the corresponding term of the word pair. In the slider scale group, the response format ranged from − 50 (e.g., *unwell*) to 50 (e.g., *well*), and in the Likert scale group, it ranged from − 2 to 2.

#### Momentary attention to and clarity of negative emotions

To measure momentary attention to and clarity of negative emotions, we used three items for each construct, which were adapted from the highest-loading items on a German trait attention to and clarity of feelings scale (Lischetzke & Eid, 2003; Lischetzke et al., 2001, 2005). The items referred to the negative emotions experienced since the last measurement occasion, and read “I have engaged with my feelings” (att1), “I have thought about how I feel” (att2), and “I have observed my feelings” (att3) for attention to negative emotions and “I have had a hard time describing my feelings” (cla1), “I have had a hard time naming my feelings” (cla2), and “I have been unsure of what I actually feel” (cla3) for clarity of negative emotions (all reverse-scored).^5^ The items were rated using an agreement format, ranging from 0 (*does not apply at all*) to 100 (*fully applies*) in the slider scale group and 1 to 5 in the Likert scale group.

#### Momentary stress

Momentary stress was assessed using a single item (similar to Erbas et al., 2019) with a unipolar five-point Likert scale ranging from 1 (*not stressed at all*) to 5 (*very stressed*). It read, “How stressed have you felt since you got up?” at the first measurement occasion per day and “How stressed have you felt since you completed the last survey?” at subsequent measurement occasions.

### Models for data analysis

We used Mplus version 8.10 (Muthén & Muthén, 1998) to compute two-level CFA models. In addition, we used R version 4.4.1 (R Core Team, 2024) for data handling and preparation. We used the R packages *misty* (Yanagida, 2024) for computing multilevel descriptive statistics and correlations, and *MplusAutomation* (Hallquist & Wiley, 2018) for handling Mplus models and output in R. The data and code used for the analyses are available online (see the Availability of Data and Materials statement).

#### Scale transformations

To compare properties such as intercepts or variances across response formats (Likert and slider scales), the items had to be on a common metric. Therefore, item scores from the slider scale format were transformed to match the range of the Likert scale format. Specifically, values from the unipolar slider scales (attention to and clarity of negative feelings), which originally ranged from 0 to 100, were rescaled to a range of 1 to 5. For example, a value of 67 on a slider scale was converted to 3.68. The values from the bipolar pleasant–unpleasant mood slider scale, originally measured on a scale of − 50 to 50, were transformed to a range of − 2 to 2.^6^

This rescaling procedure is a straightforward approach and is similar to that used by Haslbeck et al. (2025), where both response formats were normalized to the interval [0,1]. Both approaches rely on the assumption that the response formats are equidistant.

#### Two-level confirmatory factor analysis

To account for the hierarchical structure of the data, we employed a two-level confirmatory factor analysis (CFA) within the multilevel structural equation modeling framework (Asparouhov & Muthén, 2019; Muthén & Asparouhov, 2011). In this two-level CFA, which models multiple indicators of a construct measured at occasions (Level 1) nested in individuals (Level 2), the total observed-indicator covariance matrix is decomposed into a within-person (Level 1) covariance matrix and a between-person (Level 2) covariance matrix. A separate CFA model is fit to each submatrix in a single, simultaneous analysis. The Level 1 covariance matrix reflects within-person variance and random measurement error variance, whereas the Level 2 covariance matrix reflects only between-person variance (e.g., Eid et al., 2025; Muthén & Muthén, 1998).

**Fig. 2:**
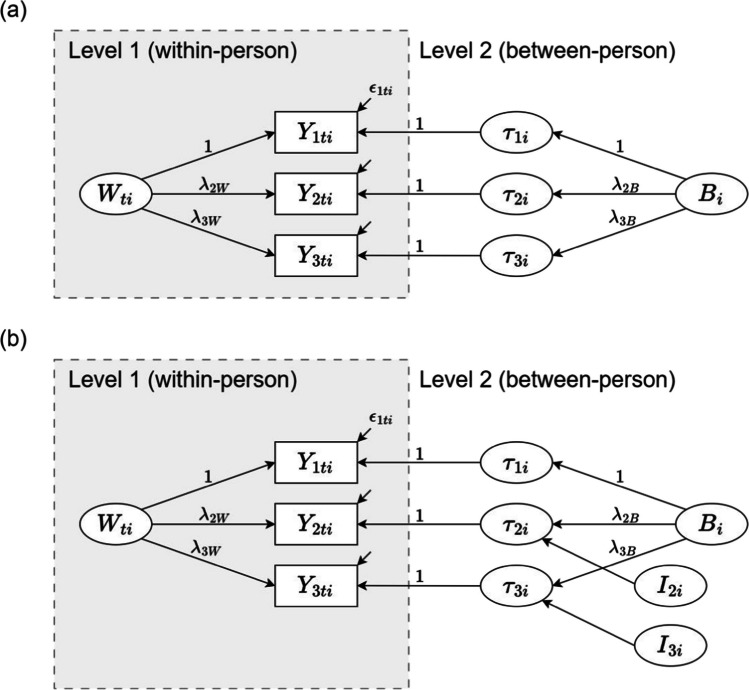
Two-level CFA models. *Note*. **a** Two-level CFA model without indicator-specific residual factors. **b** Two-level CFA model with (uncorrelated) indicator-specific residual factors. ${W}_{ti}$ = common occasion-specific latent factor. ${B}_{i}$ = common between-person latent factor. ${\tau }_{ki}$ = random intercept. ${I}_{ki}$ = indicator-specific residual factor. ${\lambda }_{kW}$ = loading (Level 1). ${\lambda }_{kB}$ = loading (Level 2). ${\epsilon }_{kti}$ = error variable. $k$ = indicator number. $t$ = occasion. $i$ = individual

Figure 2a shows a basic two-level CFA model for three observed indicators of a latent construct. The measurement equations for this model are

1 $${Y}_{kti}={\tau }_{ki}+{\lambda }_{kW}\cdot {W}_{ti}+{\epsilon }_{kti}$$

2 $${\tau }_{ki}={\alpha }_{k}+{\lambda }_{kB}\cdot {B}_{i}$$

At Level 1 (Eq. 1), an observed indicator ${Y}_{kti}$ ($k$: item, $t$: occasion, $i$: individual) is decomposed into a person-specific random intercept ${\tau }_{ki}$, a common occasion-specific latent factor ${W}_{ti}$ with loading ${\lambda }_{kW}$, and a measurement error term ${\epsilon }_{kti}$. At Level 2 (Eq. 2), the person-specific random intercept ${\tau }_{ki}$ is decomposed into a fixed intercept ${\alpha }_{k}$ and a common between-person latent factor ${B}_{i}$ with loading ${\lambda }_{kB}$.

This basic two-level CFA model implies that the indicators are perfectly homogeneous at the between-person level, meaning it assumes perfect correlations of the random intercepts at Level 2. In some applications, this assumption may be too restrictive. If the model fits the data poorly, indicator specificity at Level 2 can be accounted for by defining a reference indicator (e.g., the first indicator) and adding indicator-specific residual factors for the non-reference indicators at Level 2 (Eid et al., 2025). Figure 2b illustrates a two-level CFA model for three observed indicators, incorporating indicator-specific residual factors ${I}_{ki}$ for the non-reference indicators ($k\ne 1$) at Level 2. These residual factors capture indicator-specific variance between persons that is not shared with the reference indicator. The Level 1 equation for this model remains the same as in the basic two-level CFA model (Eq. 1), while the Level 2 equation is

3 $${\tau }_{ki}=\left\{\begin{array}{ll}{\alpha }_{k}+{\lambda }_{kB}\cdot {B}_{i},& k=1 \text{(reference indicator)}\\ {\alpha }_{k}+{\lambda }_{kB}\cdot {B}_{i}+{I}_{ki},& k\ne 1 \text{(non-reference indicators)}\end{array}\right.$$

In our application, we modeled momentary pleasant–unpleasant mood, measured by four observed indicators, using a single-factor two-level CFA model with indicator-specific residual factors at Level 2, consistent with the structure depicted in Fig. 2b. For models with multiple latent constructs, two-level CFA models can be extended to include multiple latent factors. To model momentary attention to and clarity of negative emotions, we specified a separate two-level CFA model with two latent factors at each level. In this model, we allowed covariances between the latent factors for attention and clarity at both levels and modeled (uncorrelated) indicator-specific residual factors for the non-reference indicators.^7^

Since models without indicator-specific residual factors demonstrated poor fit, we incorporated uncorrelated indicator-specific residual factors in all subsequent analyses. The original models are provided in the supplementary material.

#### Measurement invariance

To test for measurement invariance across response formats, we extended the two-level CFA models with uncorrelated indicator-specific residual factors at Level 2 to multigroup models—one for momentary pleasant–unpleasant mood, one for momentary attention to and clarity of negative emotions. Each model consisted of two groups: the Likert scale group and the slider scale group. In a first step, we specified the models with configural invariance. We then tested for equal loadings and equal error variances at the within-person level (Level 1) and equal loadings, equal intercepts, and equal variances of the indicator-specific residual factors at the between-person level (Level 2). We estimated the models using the robust maximum likelihood estimator (MLR) in Mplus.

To evaluate absolute goodness of fit, we followed the criteria proposed by Schermelleh-Engel et al. (2003). A good absolute fit is indicated by a nonsignificant ${\chi }^{2}$ test, ${\chi }^{2}$/*df* ≤ 2, comparative fit index [CFI] and Tucker–Lewis index [TLI] ≥.97, and root mean square error of approximation [RMSEA] and standardized root mean square residual [SRMR] ≤.05. An acceptable fit is indicated by a significant ${\chi }^{2}$ test with *p* ≥.01, ${\chi }^{2}$/*df* ≤ 3, CFI and TLI ≥.95, RMSEA ≤.08, and SRMR ≤.10.

To assess relative fit, we conducted ${\chi }^{2}$ difference tests and applied the cutoff criteria proposed by Chen (2007). When comparing models with and without equal factor loadings, a decrease in CFI <.005, an increase in RMSEA <.01, and an increase in SRMR <.025 indicate that the models fit relatively equally well. When comparing models with and without equal intercepts or equal error variances at Level 1 (and equal variances of the indicator-specific residual factors at Level 2), a decrease in CFI <.005, an increase in RMSEA <.01, and an increase in SRMR <.005 indicate a good relative fit.

#### Within-person and between-person reliability

We computed within-person and between-person omega coefficients for both multigroup models (i.e., one for momentary pleasant–unpleasant mood, one for momentary attention to and clarity of negative emotions) using the procedure suggested by Geldhof et al. (2014). We specified the models with configural measurement invariance and used the Mplus MODEL CONSTRAINT option to test for significant differences in the omega coefficients between the two groups.

#### Within-person variability

To estimate within-person variability, we followed the procedure proposed by Dowling et al. (2019). We specified both multigroup models (i.e., one for momentary pleasant–unpleasant mood, one for momentary attention to and clarity of negative emotions) with configural measurement invariance and tested the statistical significance of between-group differences in within-person variability of the latent variables using the MODEL CONSTRAINT option in Mplus.

#### Relationships with other time-varying variables (validity estimates)

To compare criterion validity, we tested for between-group differences in the covariances between the latent variables and the single-item measure of stress using the MODEL CONSTRAINT option in Mplus.^8^ In the model for pleasant–unpleasant mood, we compared the covariances of mood and stress across groups. In the model for attention to and clarity of negative emotions, we compared the covariances between attention and clarity, attention and stress, and clarity and stress across groups.

#### Multiple testing

Since we conducted multiple significance tests, we used the Benjamini–Hochberg procedure to control the false discovery rate (Benjamini & Hochberg, 2000).^9^ We defined all tests related to the same research question (e.g., within-person variability) as a family of tests. For the between-group comparisons of reliability, we defined the within-person and between-person reliability estimates as separate families of tests.

### Sample size considerations

The present study was part of a larger research project with multiple research questions. The project aimed for a total sample size of 330 participants (across both experimental groups) with 65 measurement occasions. Details can be found in the preregistration (see Open Practices Statement section).

To estimate statistical power for the present analyses, we conducted Monte Carlo simulations (Bolger & Laurenceau, 2013). Due to the lack of prior research on response formats in ambulatory assessment, we could not derive population parameter estimates for group differences in measurement invariance, reliability, or validity from existing data. However, we identified relevant findings from Hasselhorn et al. (2022) on group differences in within-person variability. Although their study investigated questionnaire length rather than response formats, it also included an experimental methodological manipulation. The reported effect size for differences in within-person variability in state extraversion was small (*d* =  − 0.08). We incorporated this effect size in our power analysis to ensure sensitivity to similarly small differences between response formats. The Monte Carlo simulation results indicated that a sample size of 330 participants, each completing 65 measurement occasions, provides high power (>.80, close to 1) to detect the assumed small between-group difference in within-person variability at α =.05.

## Results

### Descriptive statistics

**Fig. 3:**
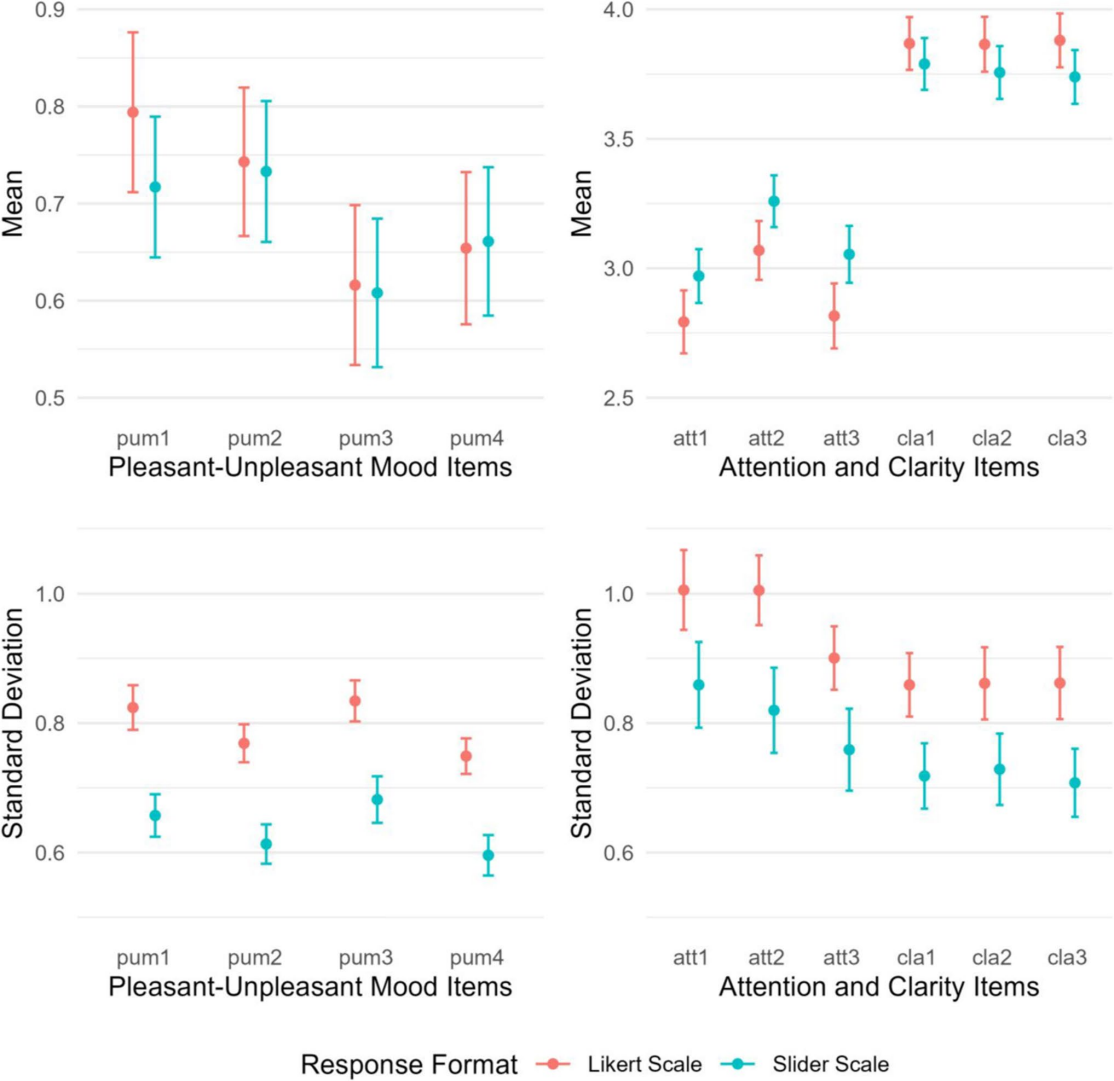
Differences in mean scores and within-person standard deviations between Likert and slider scale groups. *Note*. Mean scores and within-person standard deviations are presented for pleasant–unpleasant mood items and attention to and clarity of negative emotions items. Error bars represent 95% confidence intervals. pum = pleasant–unpleasant mood. att = attention to negative emotions. cla = clarity of negative emotions

Descriptive statistics are presented in Table 1. Additionally, item means and within-person standard deviations are visualized in Fig. 3. Across all items, within-person standard deviations were significantly higher in the Likert scale group than in the slider scale group (*M*_*SDwithin_likert*_ = 0.87, *M*_*SDwithin_slider*_ = 0.71, $\Delta$ *M*_*SDwithin*_ = 0.15, all *p* <.001). This pattern was mirrored in the intra-class correlations (ICCs), which were lower for the Likert scale items across all constructs (*M*_*ICC_likert*_ =.35, *M*_*ICC_slider*_ =.42, $\Delta$ *M*_*ICC*_ =  −.07). No systematic pattern emerged for item means. Significant differences were observed only for the attention items, with higher mean scores for slider scales compared to Likert scales (all *p* <.05).

**Table 1:** Descriptive statistics for items measuring time-varying constructs using a Likert scale or slider scale

Item	Likert scale group				Slider scale group			
	*M*	*SD*_*within*_	*SD*_*between*_	ICC	*M*	*SD*_*within*_	*SD*_*between*_	ICC
pum1	0.79	0.82	0.56	.32	0.72	0.66	0.51	.38
pum2	0.74	0.77	0.53	.32	0.73	0.61	0.50	.40
pum3	0.62	0.83	0.56	.31	0.61	0.68	0.54	.39
pum4	0.65	0.75	0.55	.35	0.66	0.60	0.55	.46
att1	2.79	1.01	0.74	.35	2.97	0.86	0.64	.36
att2	3.07	1.00	0.69	.32	3.26	0.82	0.63	.37
att3	2.82	0.90	0.79	.44	3.05	0.76	0.71	.46
cla1	3.87	0.86	0.64	.35	3.79	0.72	0.65	.45
cla2	3.87	0.86	0.66	.37	3.76	0.73	0.66	.45
cla3	3.88	0.86	0.65	.37	3.74	0.71	0.67	.48

For the mood items (pum1 to pum4), *N* at Level 1 ranged from 10,640 to 11,090 and *N* at Level 2 ranged from 200 to 206 per group. For the attention and clarity items (att1 to att3, cla1 to cla3), *N* at Level 1 ranged from 2,211 to 2,532 and *N* at Level 2 ranged from 190 to 196 per group. pum = pleasant–unpleasant mood. att = attention to negative emotions. cla = clarity of negative emotions. *SD*_*between*_ = standard deviation of individuals’ mean scores. ICC = intra-class correlation

**Fig. 4:**
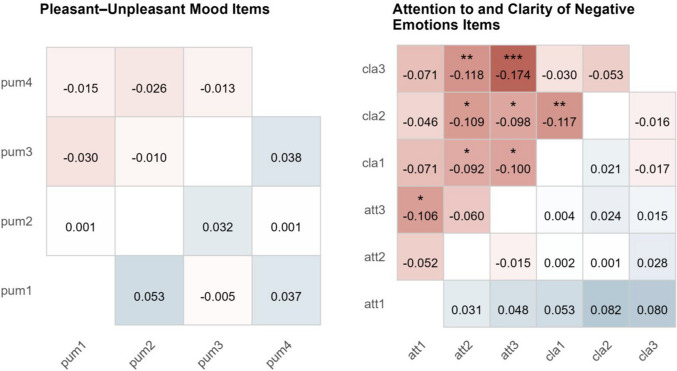
Differences in correlations among items at the within- and between-person levels between Likert and slider scale groups. *Note*. Differences represent $Co{r}_{Likert}-Co{r}_{Slider}$. Blue fields indicate positive differences, whereas red fields indicate negative differences. Differences in between-person correlations appear below the diagonal. Differences in within-person correlations appear above the diagonal. pum = pleasant–unpleasant mood; att = attention to negative emotions; cla = clarity of negative emotions. * *p* <.05. ** *p* <.01. *** *p* <.001

As shown in Fig. 4, within-person correlations were generally slightly higher in the Likert scale group than in the slider scale group (most differences |$\Delta$|< 0.1), whereas between-person correlations showed the opposite pattern (all |$\Delta$|< 0.1). A subset of the within-person differences for the attention to and clarity of negative emotions items were statistically significant. Absolute correlation values are provided in the supplemental material.

We examined whether proportions of careless (inconsistent) responses differed between the slider and Likert scale groups. In the Likert scale group, 1.14% of measurement occasions were classified as careless, compared to 2.12% in the slider scale group. A chi-square test indicated a significant difference in careless responding between groups, ${\chi }^{2}$(1) = 31.9, *p* =  <.001. The standardized effect size for this group difference was very small, $\phi =$ 0.04. That is, careless responses were slightly more frequent in the slider scale group.

### Measurement invariance across groups

Table 2 presents the absolute and relative fit indices for two sets of multigroup two-level CFA models with indicator-specific residual factors (see Two-level confirmatory factor analysis section): one for momentary pleasant–unpleasant mood and another for momentary attention to and clarity of negative emotions.^10^

**Table 2:** Model fit indices for multigroup two-level CFA models with varying degrees of measurement invariance across Likert and slider scale groups

Construct	Mod	Absolute fit							Comp	Relative fit					
		χ^2^	scf	*df*	*p*	CFI	RMSEA	SRMR		Δχ^2^	Δ*df*	*p*_Δ_	ΔCFI	ΔRMSEA	ΔSRMR
PUM	1	339.85	2.13	10	<.001	0.979	0.055	0.041	–	–	–	–	–	–	–
	2	322.15	2.27	13	<.001	0.980	0.047	0.042	1	2.82	3	.421	0.001	− 0.008	0.001
	3	645.46	4.12	17	<.001	0.960	0.058	0.080	2	190.09	4	<.001	− 0.020	0.011	0.038
	4	351.54	2.08	16	<.001	0.979	0.044	0.045	2	1.96	3	.580	− 0.001	− 0.003	0.003
	5	381.04	2.00	19	<.001	0.977	0.042	0.049	4	18.98	3	<.001	− 0.002	− 0.002	0.004
	6	396.24	2.00	22	<.001	0.976	0.040	0.043	5	15.88	3	.001	− 0.001	− 0.002	− 0.006
ATT/CLA	1	135.06	1.21	36	<.001	0.980	0.034	0.032	–	–	–	–	–	–	–
	2	143.40	1.26	40	<.001	0.979	0.033	0.034	1	10.11	4	.039	− 0.001	− 0.001	0.002
	3	445.12	1.66	46	<.001	0.918	0.060	0.086	2	128.66	6	<.001	− 0.061	0.027	0.052
	4	146.18	1.24	44	<.001	0.979	0.031	0.034	2	1.12	4	.891	0.000	− 0.002	0.000
	5	151.45	1.23	48	<.001	0.979	0.030	0.034	4	4.19	4	.380	0.000	− 0.001	0.000
	6	147.68	1.28	52	<.001	0.980	0.028	0.034	5	1.50	4	.826	0.001	− 0.002	0.000

The varying degrees of measurement invariance across groups were as follows: Model 1 = no equality constraints; Model 2 = equal loadings at Level 1; Model 3 = equal loadings and equal error variances at Level 1; Model 4 = equal loadings at Level 1 and Level 2; Model 5 = equal loadings at Level 1 and Level 2, equal intercepts at Level 2; Model 6 = equal loadings at Level 1 and Level 2, equal intercepts, and equal variances of indicator-specific residual factors at Level 2. PUM = pleasant–unpleasant mood. ATT/CLA = attention and clarity. Mod = model. Comp = comparison model. scf = scaling correction factor for χ^2^ (MLR-estimator)

Each set included six models. For mood, both the unconstrained model (Model 1) and the model with equal loadings at Level 1 across groups (Model 2) showed good absolute fit (and good relative fit for Model 2 compared to Model 1). However, model fit deteriorated when Level 1 error variances were also constrained across groups (Model 3). Examination of model misfit indicated that the Likert scale group had larger error variances, consistent with its descriptively larger within-person item-level standard deviations (see Descriptive statistics). Consequently, we constrained only the Level 1 loadings (but not Level 1 error variances) across groups before proceeding to test measurement invariance at the between-person level.

Building on this specification, we added equality constraints at Level 2—equal loadings (Model 4) and equal loadings plus equal intercepts (Model 5)—both of which demonstrated good fit. Finally, Model 6, which constrained loadings at Level 1 and loadings, intercepts, and variances of the indicator-specific residual factors at Level 2, also fit the data well. Thus, Model 6 was retained as the final model for momentary pleasant–unpleasant mood.

The same pattern emerged for momentary attention to and clarity of negative emotions: between-group differences were found in in Level 1 error variances, but measurement invariance held across groups for other parameters. Accordingly, Model 6 was also selected as the final model for these constructs.

### Latent means

Since the restrictions in the selected models met the criteria for strong measurement invariance (equal loadings and intercepts across groups), we compared the constructs’ latent means between groups. We set the latent means in the Likert scale group to 0, so the latent mean estimates in the slider scale group reflect between-group differences. To control the false discovery rate, we applied the Benjamini–Hochberg procedure (Benjamini & Hochberg, 2000). No significant mean differences were found in mood ($\Delta M$ = − 0.06, ${p}_{adj}$ =.245) or clarity ($\Delta M$ = − 0.1, ${p}_{adj}$ =.214). However, we found a significant difference for attention ($\Delta M$ = 0.20, ${p}_{adj}$ =.033).

### Reliability

Omega coefficients estimated using the approach proposed by Lai (2021) are shown in Table 3. At the within-person level, omega values for mood were very high in both groups (>.89). Omega values for attention and clarity were high across both groups (>.73), considering that each construct was assessed with only three items. Descriptively, the slider scale group had higher within-person omega values across all constructs than the Likert scale group. However, the between-group differences in within-person omega (${\omega }_{Likert}-{\omega }_{Slider}$) were negligible for mood ($\Delta \omega =$− 0.007) and relatively small for attention ($\Delta \omega =$− 0.049) and clarity ($\Delta \omega =$− 0.059). None of these differences were statistically significant following false discovery rate correction.

**Table 3:** Within- and between-person omega coefficients

Level	Construct	$\upomega$_*Likert*_	$\upomega$_*Slider*_	Δ $\upomega$	*p*
Within	PUM	0.895	0.902	− 0.007	.409
	ATT	0.739	0.788	− 0.049	.065
	CLA	0.732	0.791	− 0.059	.026
Between	PUM	0.970	0.960	0.010	.022
	ATT	0.858	0.869	− 0.011	.467
	CLA	0.882	0.893	− 0.010	.129

Omega coefficients were calculated using the method by Lai (2021). No statistically significant differences between response formats were detected at either level after controlling the false discovery rate using the Benjamini–Hochberg procedure. *PUM* = pleasant–unpleasant mood. *ATT* = attention to negative emotions. *CLA* = clarity of negative emotions

**Fig. 5:**
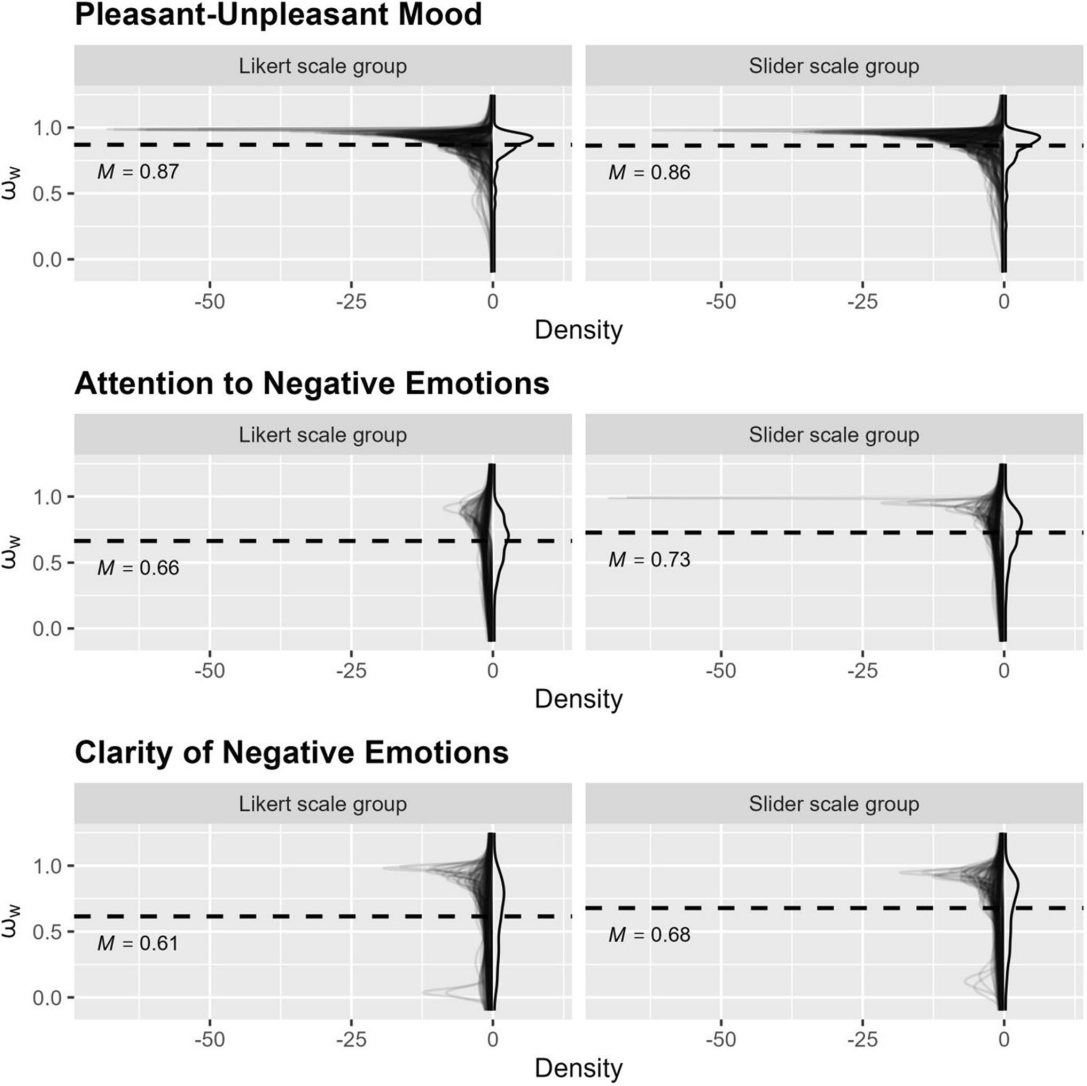
Within-person reliability estimates based on the two-level random dynamic model (2RDM). *Note*. Density plots of within-person reliability estimates (ω_w_) for momentary pleasant–unpleasant mood, attention to negative emotions, and clarity of negative emotions in the Likert scale and slider scale groups. Dashed horizontal lines represent group means

Within-person reliability estimates derived from the 2RDM framework and their respective means are shown in Fig. 5. For mood, the between-group difference in omega was small and not statistically significant ($\Delta \Omega =$ 0.007, *p* =.510). For attention ($\Delta \Omega =$ −0.063, *p* =  <.001) and clarity ($\Delta \Omega =$ −0.067, *p* =.012), within-person omega was higher in the slider scale group. These findings remained unchanged after false discovery rate correction. Overall, there was no consistent pattern of differences in reliability between response formats across the two estimation frameworks.

Between-person omega coefficients were very high across groups and constructs (>.85). For mood, reliability was slightly higher in the Likert scale group ($\Delta \omega =$ 0.01). For attention ($\Delta \omega =$− 0.011) and clarity ($\Delta \omega =$− 0.01), reliability was slightly higher in the slider scale group. However, none of the between-group differences remained statistically significant after false discovery rate correction.

### Within-person variability

Within-person variability estimates for the three constructs are presented in Table 4. Descriptively, the Likert-scale group showed larger within-person variability across all constructs. However, the between-group difference was significant only for mood.

**Table 4:** Within-person variability estimates for the Likert and slider scale groups

Construct	*Var*_*Likert*_	*Var*_*Slider*_	*ΔVar*	*SD*_*Likert*_	*SD*_*Slider*_	*ΔSD*	*p*
PUM	0.428	0.277	0.151	0.654	0.526	0.128	** <.001**
ATT	0.398	0.362	0.036	0.631	0.602	0.029	.613
CLA	0.310	0.261	0.049	0.557	0.511	0.046	.337

Group differences that remained significant after false discovery rate correction (Benjamini & Hochberg, 2000) are indicated in boldface. *PUM* = pleasant–unpleasant mood. *ATT* = attention to negative emotions. *CLA* = clarity of negative emotions. *Likert* = Likert scale group

### Validity

Covariances between the latent constructs and the single-item measure of stress are shown in Table 5. Descriptively, neither response format consistently produced larger or smaller covariances. None of the between-group differences were statistically significant.

**Table 5:** Criterion validity estimates: within-person relationships with momentary stress for the Likert and slider scale groups

Construct	*Cov*_*Likert*_	*Cov*_*Slider*_	Δ*Cov*	*p*	*Cor*_*Likert*_	*Cor*_*Slider*_	Δ*Cor*
PUM with stress	− 0.30	− 0.24	− 0.05	.028	− 0.49	− 0.48	0.01
ATT with stress	− 0.01	0.01	− 0.03	.330	− 0.02	0.03	0.05
CLA with stress	− 0.01	− 0.02	0.01	.673	− 0.01	− 0.03	− 0.02
ATT with CLA	− 0.03	0.03	− 0.06	.014	− 0.07	0.11	0.18

*p*-values represent the significance of differences in covariance estimates across the Likert and slider scale groups. No statistically significant differences between response formats were detected after controlling the false discovery rate using the Benjamini–Hochberg procedure. *PUM* = pleasant–unpleasant mood. *ATT* = attention to negative emotions. *CLA* = clarity of negative emotions. *Cov* = covariance. *Cor* = correlation

## Discussion

The main aim of this study was to compare the psychometric properties of Likert and slider scales in ambulatory assessment. We examined three time-varying constructs: pleasant–unpleasant mood, and momentary attention to and clarity of negative emotions. To ensure that any differences observed were due to the response format itself, we experimentally manipulated the response format between participants. To test measurement invariance and compare reliability, within-person variability, and criterion validity across response formats, we conducted multigroup two-level CFA models.

The results revealed only marginal differences between the two response formats. Factor loadings at the within- and between-person levels, intercepts, and indicator-specific residual factor variances at the between-person level did not differ across groups, allowing for valid comparisons of latent means and covariances across response formats. Only error variances at the within-person level were higher in the Likert scale than in the slider scale group. Similarly, the Likert scale group exhibited higher within-person variability for mood. This pattern is further reflected in its descriptively lower intraclass correlations and higher within-person standard deviations. We found no significant differences in reliability or criterion validity between the response formats.

Several factors may explain the significantly higher within-person variability in mood in the Likert scale group relative to the slider scale group. One explanation for this difference is the discrete nature of the Likert scale: Participants wishing to indicate a small change in mood between measurement occasions had to select the next available response option, representing a relatively larger step than the minimal change possible on the continuous slider scale. This could have led to greater within-person variability and larger error variances at Level 1 in the Likert scale group. Another possibility is that participants were more careless when using slider scales, leading to more uniform responses. However, this is unlikely, as we found no significant differences in reliability between response formats. Findings from cross-sectional studies suggest limited benefits of Likert scales with more than five response options. A few studies report slight improvements in reliability and validity when increasing from five- to six- or seven-point scales, but no additional gains beyond seven points (Simms et al., 2019). Other studies found no improvements in reliability and validity beyond a five-point scale (Donnellan & Rakhshani, 2023; Rakhshani et al., 2024). However, these studies did not directly compare variability of response distributions.

Finally, we found that careless (inconsistent) responding was more prevalent in the slider scale group than the Likert scale group. However, this effect was very small. This suggests that response format may influence participant engagement or attentiveness during ambulatory assessments, an aspect warranting further investigation.

Overall, our results suggest that Likert and slider scales yield largely comparable psychometric properties in ambulatory assessment studies, with no clear advantage for either response format, except for a slightly higher incidence of careless responding observed in the slider scale group.

When comparing our findings with those of Haslbeck et al. (2025) on similar outcomes, several differences emerge. Whereas Haslbeck et al. (2025) reported higher mean levels for slider scales, we observed no systematic differences in item means between the two response formats, with the exception of the attention items. In contrast to Haslbeck et al. (2025), who found no differences in within-person standard deviations, we observed significantly larger within-person standard deviations for Likert scales across all items. Additionally, we were unable to replicate their finding of consistently higher criterion correlations for slider scales. However, the pattern for within-person item correlations was consistent with that reported by Haslbeck et al. (2025), with descriptively higher correlations for slider scales and a subset of differences yielding statistically significant results. Taken together, our results show both similarities and discrepancies with the findings of Haslbeck et al. (2025). These differences may be attributable to variations in study design, sample characteristics, or the specific implementation of the response formats. Such variation underscores the need for further research to clarify the conditions under which response format influences descriptive statistics in ambulatory assessment data.

### Limitations

While this study provides valuable insights into the psychometric properties of Likert and slider scales in ambulatory assessment, some limitations of the present study should be considered when interpreting the findings.

First, due to the negative emotion filter item (“Have you experienced negative emotions since you completed the last survey?”), the sample size for the attention to and clarity of negative emotions models was considerably smaller than for the mood models. Although both sample sizes were sufficient to achieve high power for detecting small differences in within-person variability, the inability to analyze all three constructs in a single three-factor CFA model without significant data loss was suboptimal. A three-factor CFA model would have enabled additional latent covariance comparisons relevant to criterion validity (e.g., mood and attention).

Second, our validity estimates could be improved in future studies. The single-item stress measure was always presented as a Likert scale, potentially introducing shared method variance with the Likert-based constructs. As a result, true covariances cannot be distinguished from shared method variance. Future studies could implement a multitrait-multimethod (MTMM) design to better differentiate between shared method variance and true covariances between constructs.

Third, the Likert scales in this study had labels only at the endpoints to match the corresponding slider scales. However, it is generally recommended that Likert scales have labels for all response options (Menold & Bogner, 2016). Future research could examine whether our findings generalize to Likert scales with more than two labels.

Finally, these results may not generalize to different populations, such as children, adolescents, or clinical groups.

### Future directions

As a direct continuation of this research, a possible next step is to conduct a similar experimental ambulatory assessment study using a Likert scale with six or seven response options. Additionally, varying the number of Likert scale response options within an ambulatory assessment study could provide further insights into differences in within-person variability. This could be combined with an MTMM design to compare covariances between response formats.

Another aspect for future research concerns the rescaling procedure. Our approach, as well as that of Haslbeck et al. (2025), relies on the assumption that both response formats are equidistant. To our knowledge, only one study has empirically investigated this issue, reporting differences in the perceived distances between points on a visual analogue scale and a Likert scale (García-Pérez, 2023). Future research could explore alternative transformation methods that better account for potential non-equidistance in Likert scale responses.

In this study, we analyzed measurement invariance across response formats as a between-subject factor. However, another important issue is measurement invariance over time (Millsap, 2010; Widaman et al., 2010). An interesting research question would be to compare measurement invariance over time for the two response formats. One approach could involve cross-classified models with dynamic structural equation modeling to analyze factor loadings and intercepts over time (Asparouhov & Muthén, 2016; Asparouhov et al., 2018; McNeish et al., 2021). However, measurement invariance over time in such models remains underexplored, and open questions remain, including the potential causes of non-invariance (Barta et al., 2012; McNeish et al., 2021).

Another important domain in which Likert and slider scales should be compared is response styles. Recent work by Henninger et al. (2025) showed that extreme response style can bias estimates of affect dynamics in intensive longitudinal data. However, as extreme response style in that study was computed from Likert scale responses, it remains unclear whether participants exhibit similar response-style tendencies when using slider scales. Future research should therefore examine whether response styles are stable across response formats or vary as a function of the response format employed.

## Conclusion

Overall, neither response format appears to offer a clear advantage over the other in ambulatory assessment studies. So, how should researchers decide which response format to use? One argument for using Likert scales is their extensive use and validation in behavioral and social sciences. Most instruments have been designed and tested with Likert scales, making them a well-established choice. Although our findings suggest that Likert and slider scales are largely comparable, we recommend that any modifications to an existing instrument’s response format be made with careful consideration.

On the other hand, slider scales may be preferable due to their intuitive usability, particularly on touchscreens. Furthermore, in long surveys with many Likert scale items, slider scales may provide a welcome variation for participants. However, it is important to note that, despite producing continuous data, slider scales do not necessarily yield more precise measurements than Likert scales. In conclusion, both response formats are valid choices, but researchers should carefully weigh the advantages and limitations of each format when designing their studies.

## Data Availability

Data and supplementary materials are available at 10.17605/OSF.IO/THDF5. All research questions and the study’s design and its analysis were preregistered on the OSF in the same repository.

## Footnotes

1 The preregistered reliability estimation procedure following Geldhof et al. (2014) is documented in the supplementary materials.

2 Because of a programming error, the momentary stress item was displayed with minor differences from the planned response format. Specifically, in the Likert scale group, the item was mistakenly presented as a Likert scale instead of a slider scale (opposite to the manipulation for other items). To address this, data from a third experimental group (*N* = 93) were collected using the intended response format. However, because this group does not meet the sample size criteria from our power analysis, it is not included in the present study.

3 A total of 341 participants completed the initial survey for the study but did not proceed with the ambulatory assessment phase. This dropout rate was largely due to technical issues with the software (SEMA3), which was unavailable for several months on newly released Android versions in 2023.

4 As an operationalization of inconsistent responding, we constructed a psychometric antonyms index for each measurement occasion using bipolar momentary mood item pairs, following the conceptual approach described by Meade and Craig (2012). These pairs captured three mood dimensions: pleasant–unpleasant (*unwell–well*, *good–bad*, *dissatisfied–satisfied*, *happy–unhappy*), calm–tense (*tense–relaxed*, *calm–restless*), and wakefulness–tiredness (*tired–awake*, *rested–sleepy*). Inconsistent responding was defined as an “illogical” response pattern within a mood item pair—that is, providing extreme responses in the same direction on both semantically opposite items. For example, in the slider scale group (range, –50 to 50), responses were classified as inconsistent when both items of a pair had values ≤ –30 or ≥ 30 (e.g., rating both *tense–relaxed* and *calm–restless* near + 40). In the Likert scale group (range, –2 to 2), this corresponded to both responses being –2 or 2 (e.g., strongly agreeing with both *happy–unhappy* and *unwell–well*).

5 At each measurement occasion, participants were asked whether they had experienced negative emotions since the last measurement occasion. If they had, participants rated their negative emotional experience and subsequently responded to the momentary attention to and clarity of negative emotions items. If they had not, filler items regarding activities were presented to ensure that the number of items remained consistent across measurement occasions. Negative emotions were reported at *n* = 4,743 measurement occasions (22%) nested in 386 participants. Consequently, analyses of attention to and clarity of negative emotions were based on this smaller subsample. A power analysis confirmed that this sample size was sufficient to detect the effect size specified in our initial power analysis (see Sample size considerations section) with a power of.97 (at α = 5%).

6 In the preregistration, we stated that we would instead rescale the Likert scales to match the metric of the slider scales. However, this approach led to estimation problems in Mplus due to excessively large values. Mathematically, the results are equivalent regardless of the transformation direction.

7 We originally planned to specify a three-dimensional model with all constructs (see preregistration in Open Practices Statement section). However, due to the different sample sizes of the pleasant–unpleasant mood and attention to and clarity of negative emotion items, we decided to specify separate models.

8 We tested for differences in covariances and not in correlations, because in regression analysis one would also compare the unstandardized and not the standardized regression coefficients (e.g., Cohen, 2013).

9 The false discovery rate (FDR) is the expected proportion of false positives among all tests declared significant. The Benjamini–Hochberg (BH) procedure controls the FDR by comparing each ordered *p* value to a threshold that depends on its rank among all tests. Specifically, if the *p* values are sorted in ascending order ${p}_{\left(1\right)}\le {p}_{\left(2\right)}\le \dots \le {p}_{\left(m\right)}$, the BH critical value for the $i$ th test is $\frac{i}{m}\times q$, where $m$ is the total number of tests and $q$ is the desired FDR level (e.g., 0.05). The largest $i$ for which ${p}_{\left(i\right)}\le \frac{i}{m}\times q$ is considered significant, and all tests with smaller *p* values are also deemed significant.

10 As preregistered, we first specified single-factor models for attention and for clarity. Since both models demonstrated good fit, we proceeded to the two-factor models. Model fit indices for the single-factor models can be found in the supplementary material.

## Code availability

The analysis code is available in the supplementary material at 10.17605/OSF.IO/THDF5.

## Declarations

### Open practices statement

The research questions, study design, and data analysis plan were preregistered on the Open Science Framework (10.17605/OSF.IO/THDF5). The data and analysis scripts are available in the same OSF project.
